# Self-reported cardiovascular disease risk factor screening among people living with HIV vs. members of the general population in Botswana: a community-based study

**DOI:** 10.1186/s12889-024-17651-6

**Published:** 2024-01-16

**Authors:** Onkabetse Julia Molefe-Baikai, Kago Kebotsamang, Pinkie Modisawakgomo, John Thato Tlhakanelo, Keneilwe Motlhatlhedi, Thato Moshomo, Nabila Farah Youssouf, Tiny Masupe, Tendani Gaolathe, Neo Tapela, Shahin Lockman, Mosepele Mosepele

**Affiliations:** 1 Faculty of Medicine, Department of Internal Medicine, University of Botswana, Princess Marina Hospital, Gaborone, Botswana; 2 Faculty of Social Sciences, Department of Statistics, University of Botswana, Gaborone, Botswana; 3 Botswana Harvard AIDS Institute Partnership, Gaborone, Botswana; 4 Faculty of Medicine, Department of Family Medicine and Public Health, University of Botswana, Gaborone, Botswana; 5 London School of Hygiene and Tropical Medicine, London, UK; 6 Department of Immunology and Infectious Diseases, Harvard T. H. Chan School of Public Health, Boston, USA; 7 International Consortium for Health Outcomes Measurement, Boston, USA; 8 Division of Global Health Equity, Brigham and Women’s Hospital, Boston, USA; 9 Division of Infectious Diseases, Brigham and Women’s Hospital, Boston, USA

**Keywords:** Cardiovascular disease risk factors, HIV, Screening, PLWH, Botswana

## Abstract

**Background:**

Morbidity and mortality due to cardiovascular diseases (CVDs) are high and increasing in low- and middle-income countries. People living with HIV (PLWH) are more likely to experience CVD than members of the general population. Therefore, we aimed to assess whether PLWH were more likely to have previously been screened for cardiovascular disease risk factors (CVDRFs) than people without HIV.

**Methods:**

A population-based, cross-sectional study was conducted among individuals aged 16 to 68 years across 22 communities in Botswana from February to August 2017 as part of a larger community-based cluster randomized HIV treatment-as-prevention trial. Participants were asked if they had been screened for and counselled on cardiovascular disease risk factors (history of hypertension or blood pressure check, blood glucose and cholesterol measurements, weight check and weight control, tobacco smoking and cessation, alcohol use and physical activity) in the preceding 3 years. HIV testing was offered to those with an unknown HIV status. Multiple logistic regression analysis controlling for age and sex was used to assess the relationship between CVDRF screening and HIV status.

**Results:**

Of the 3981 participants enrolled, 2547 (64%) were female, and 1196 (30%) were PLWH (93% already on antiretroviral therapy [ART]). PLWH were more likely to report previous screening for diabetes (25% vs. 19%, *p* < 0.001), elevated cholesterol (17% vs. 12%, *p* < 0.001) and to have had their weight checked (76% vs. 55%, *p* < 0.001) than HIV-uninfected participants. PLWH were also more likely to have received counselling on salt intake (42% vs. 33%, *p* < 0.001), smoking cessation (66% vs. 46%, *p* < 0.001), weight control (38% vs. 29%, *p* < 0.001), physical activity (46% vs. 34%, *p* < 0.001) and alcohol consumption (35% vs. 23%, *p* < 0.001) than their HIV-uninfected counterparts. Overall, PLWH were more likely to have received screening for and/or counselling on CVDRFs (adjusted odds ratio 1.84, 95% CI: 1.46–2.32, *p* < 0.001).

**Conclusion:**

PLWH were almost two times more likely to have been previously screened for CVDRFs than those without HIV, indicating a need for universal scale-up of integrated management and prevention of CVDs in the HIV-uninfected population.

## Background

Cardiovascular diseases (CVDs) are a worldwide epidemic with a rapid increase in middle- and low-income countries [1, 2]. CVD accounted for one-third of all global deaths in 2015, and there were an estimated 422 million prevalent CVD cases in the same year [3]. In 2012, CVD was the leading cause of noncommunicable disease (NCD) deaths among people under the age of 70 years globally (37% of NCD deaths) [4]. According to the world health organization (WHO) CVD account for 17.9 million deaths annually and remains the leading cause of NCD deaths in 2023 [5].

The global burden of cardiovascular disease attributable to HIV infection has also increased. Several studies have demonstrated an excess risk of cardiovascular diseases, including myocardial infarction, ischaemic stroke, pulmonary hypertension, and heart failure, among people living with HIV (PLWH) [6–12]. A recent meta-analysis of the burden of HIV-associated CVD demonstrated a twofold higher likelihood of developing CVD among PLWH than among HIV-uninfected individuals [13]. Since 1990, the rate and risk of cardiovascular disease among PLWH as well as the disability-adjusted life-years (DALYs) from HIV-associated CVD have been steadily rising, with the global population incidence rising threefold from 0.36% in 1990 to 0.92% in 2015 [13].

The highest HIV-attributable CVD burden has been reported in the sub-Saharan countries of Swaziland, Lesotho, Botswana and South Africa, where HIV accounts for more than 15% of the CVD burden [13]. Sub-Saharan Africa is experiencing a rise in CVD burden attributable to the increasing prevalence of traditional CVD risk factors (CVDRFs), the persistence of infectious causes of heart disease and the ageing HIV population with accompanying chronic comorbidities such as CVD [14–20].

CVDRFs tend to increase with age and often occur in clusters [21–26]. The Botswana STEPS survey on the key risk factors for NCD found that most adults have at least one of the five major risk factors for CVD, namely, current daily smoking, eating fewer than five servings of fruits and vegetables per day, a low level of physical activity, being overweight, and having elevated blood pressure [26].

Considering the potential clustering of CVDRFs, it is imperative that screening for CVDRFs be performed during routine adult care. Guidelines on the primary prevention of cardiovascular diseases recommend routine screening, counselling and management of cardiovascular risk factors [27–29]. The Botswana multisectoral strategy for the prevention of NCDs includes the integration of NCD interventions and early screening for CVD at the primary health care level, with an aim of as much as 80% of type 2 diabetes being treated at the primary health care level [30–32].

Despite the guideline recommendation of CVD integration in primary care and HIV care settings [31, 32], there are limited data in Botswana regarding the extent of screening for CVDRFs among both PLWH and HIV-uninfected individuals. The current study therefore aimed to assess the proportion of the population who had been screened for and counselled on CVDRFs by health care workers and to assess whether there was a difference in screening coverage based on HIV status. In this way, this study sought to advance knowledge on the practices of health care personnel regarding CVDRF screening as well as health counselling in a population with a high HIV prevalence. This study will further help to inform quality improvement strategies for early identification and better management of and subsequent reduction in CVD risk factors.

## Methods

### Study setting, study design and participants

This study on CVDRFs was nested in a large on-going community-based cluster randomized trial that took place in 30 rural and peri-urban communities in Botswana (the Botswana Combination Prevention Project or BCPP). The BCPP was aimed at assessing the effect of a combination prevention package (including HIV treatment-as-prevention) on HIV incidence [33]. Consenting individuals aged 16–68 years in a simple random sample of approximately 20% of all households in the participating communities took part in the BCPP survey cohort and were followed up from 2013 to 2018.

During the final BCPP “20% household” survey cohort visit (from February 2017 to August 2017), we enrolled individuals in 22 of the 30 communities in a nested sub-study of CVDRFs and hypertension by convenience sampling. Screening for and counselling on CVDRFs was assessed at the final visit of the parent trial. Only citizens of Botswana were eligible to participate in this study.

### Data collection and materials

A trained study interviewer administered a CVDRF assessment and counselling survey questionnaire developed for this study. The questionnaire consisted of closed-ended questions and was supplemented with a review of participants’ medical documents where available and measurement of blood pressure (twice, at least 5 min apart) [34] and waist/hip circumference as per the World Health Organization guidelines [35]. The questionnaire was translated to Setswana (Setswana and English being the two national languages in the country) and back translated to English to ensure consistency in meaning. Participants were asked if a health care worker had asked about or performed any of the following in the preceding 3 years: history of hypertension or blood pressure check, blood glucose measurement, blood cholesterol measurement, weight check and weight control, tobacco smoking and smoking cessation, alcohol use and moderate physical activity. Information on the participant’s HIV status was obtained by word of mouth and confirmed from medical records. HIV testing was offered to those with an unknown HIV status, and those found to be HIV-infected were referred to a local health facility for further management. The diagnosis of hypertension was based either on the presence of treated hypertension (current receipt of any antihypertensive) regardless of the current blood pressure (BP) *or* systolic BP ≥ 140 and/or diastolic blood pressure ≥ 90 mmHg (as measured during the study visit).

### Statistical analysis

Statistical analyses were conducted using R statistical software (version 4.2.2, R Core Team 2022). Demographic and clinical characteristics were summarized by performing a standard descriptive analysis [percentages, medians and interquartile range (IQR)]. The chi-square test for independence was used to assess the association between categorical variables, whereas the Wilcoxon rank sum test was used to compare the medians of the continuous variables by HIV status. Logistic regression was performed to assess the relationship between HIV status and screening for/counselling on CVDRFs. Multiple logistic regression was then performed to adjust for age and sex. A p value less than 0.05 was considered statistically significant.

## Results

We included 3981 participants in this study, of whom 2547 (64%) were female. The median age (IQR) of PLWH [42(35–50)] was significantly higher than that of HIV-uninfected people [32(24–46)]. 30% (1196 out of 3981) of the participants were PLWH, the vast majority (93%) of whom were on antiretroviral therapy. HIV-uninfected participants were more likely to have a secondary level education or higher (73% vs. 54%, *p* < 0.001) than PLWH.

Cigarette smoking was generally low among the participants, with only 534 (13%) actively smoking cigarettes. PLWH had a higher waist-to-hip ratio than HIV-uninfected participants, with a median (IQR) waist-to-hip ratio of 0.85 (0.80–0.92) vs. 0.84 (0.78–0.91, *p* = 0.006) for females and 0.88 (0.83–0.95) vs. 0.85 (0.81–0.93, *p* = 0.009) for males, respectively.

The participants’ characteristics stratified by HIV status are summarized in Table 1.

**Table 1:** Demographic and clinical characteristics of the participants

Characteristics	All *N* = 3981	HIV infected *N* = 1196^*^	HIV uninfected *N* = 2764	P value
*Age, median (IQR)*	35 (26, 48)	42 (35, 50)	32 (24, 46)	< 0.001
16–24, N (%)	647 (16)	42 (4)	599 (22)	
25–34, N (%)	1 131 (28)	202(17)	921 (33)	
35–44, N (%)	905 (23)	416 (35)	485 (18)	
45–54, N (%)	620 (16)	316 (26)	303 (11)	
55–68, N (%)	678 (17)	320 (18)	456 (16)	
Sex				< 0.001
*Female, N (%)*	2 547 (64)	879 (73)	1 657 (60)	
*Education level* ^*#*^				< 0.001
Non-formal, N (%)	467 (12)	181 (15)	286 (10)	
Primary, N (%)	836 (21)	373 (31)	461 (17)	
Secondary, N (%)	2 075 (52)	568 (48)	1 497 (54)	
Higher than secondary N (%)	585(15)	67(6)	509(19)	
*Cigarette smoking*				
Yes, N (%)	534 (13)	163 (14)	368 (13)	0.800
*Blood Pressure, mmHg*				
Systolic, median (IQR)	119 (109, 130)	117 (108, 128)	119 (109,130)	0.011
Diastolic, median (IQR)	80 (72, 88)	79.5 (72, 88)	80 (72, 88)	0.659
*Waist-to-Hip Ratio*				
*Female, Median (IQR)*	0.84 (0.79, 0.91)	0.85 (0.80,0.92)	0.84 (0.78, 0.91)	0.006
*Male, Median (IQR)*	0.86 (0.81, 0.93)	0.88 (0.83,0.95)	0.85 (0.81, 0.93)	0.009
*Currently prescribed antihypertensive medication*				
Yes, N (%)	350 (9)	112 (9)	237 (9)	0.428
No, N (%)	3 631 (91)	1 084 (91)	2 527 (91)	
Median CD4 count, cells/ul, (IQR)	N/A	542.5 (402,730)	N/A	N/A
ART status				
On ART, N (%)	N/A	1 110 (93)	N/A	N/A
ART-naïve, N (%)	N/A	70 (6)	N/A	N/A
ART defaulter, N (%)	N/A	16 (1)	N/A	N/A

^*^21 Missing HIV status ^#^18 Missing education level

### Screening and counselling for cardiovascular disease risk factors

Despite 71% of all participants having been screened for or counselled on at least one CVDRF, screening for or counselling on individual CVDRFs was generally low. PLWH were more likely than HIV-uninfected participants to be screened for and counselled on each CVDRF that we inquired about (Table 2). The total number of CVDRFs screened for per respondent was also compared between PLWH and HIV-uninfected individuals. PLWH were again more likely to be screened for or counselled on more CVDRFs with a median (IQR) of 3 (1–5) screened factors compared to a median of 1 (0–4) screened factor for their HIV-uninfected counterparts.

**Table 2:** Screening and counselling coverage for CVD risk factors by HIV status

Counselling/Screening	ALL n (%)	HIV Infected n (%)	HIV Uninfected n (%)	p value
Diabetes screening	838 (21.0)	299 (25.0)	536 (19.4)	< 0.001
Cholesterol screening	536 (13.5)	201 (16.8)	332 (12.0)	< 0.001
Weight checked in preceding 3 years	2 426 (60.9)	904 (75.6)	1 510 (54.6)	< 0.001
Salt intake counselling	1 426 (35.8)	505 (42.2)	916 (33.1)	< 0.001
Smoking cessation counselling	275 (51.5)	107 (65.6)	168 (45.7)	< 0.001
Weight control counselling	1 260 (31.7)	453 (37.9)	802 (29.0)	< 0.001
Physical activity counselling	1 486 (37.3)	555 (46.4)	926 (33.5)	< 0.001
Alcohol counselling	1 050 (26.4)	420 (35.1)	627 (22.7)	< 0.001
**One or more CVDRFs** **Screened for**	2 841 (71.4%)	975 (81.5)	1 851 (67.0)	< 0.001
Total CVDRFs screened for*	2 (0–4)	3 (1–5)	1 (0–4)	< 0.001

* The Wilcoxon rank sum test was used to compare the medians

We evaluated several demographic and clinical characteristics as individual (univariate) predictors of screening for/counselling on at least one CVDRF (Table 3). We found that having HIV and hypertension and older age were all associated with being screened for/counselled on at least one CVDRF.

**Table 3:** Univariate analysis of factors associated with screening and counselling for at least one CVDRF

	Screened/Counselled for 1 or more risk factors		
	n (%)	Unadjusted odds ratio (95% CI)	P value
HIV Status*			
Negative (ref)	1 851 (67)	reference	
Positive	975 (82)	2.17 (1.84–2.57)	< 0.001
**Hypertension**			
None (ref)	1 449 (72)	reference	
Hypertensive	605 (79)	1.47 (1.21–1.80)	< 0.001
Sex*			
Female (ref)	1 932 (76)	reference	
Male	909 (63)	0.55 (0.48–0.63)	< 0.001
Age*			
16–24* (ref)	370 (13)	reference	
25–34	782 (28)	1.67 (1.37–2.05)	< 0.001
35–44	678 (24)	2.23 (1.80–2.78)	< 0.001
45–54	484 (17)	2.66 (2.08–3.41)	< 0.001
55–68	527 (18)	2.61 (2.06–3.32)	< 0.001
**Level of Education**			
Non-formal (ref)	350 (74.9)	reference	
Primary	636 (76.1)	1.06 (0.82–1.38)	0.648
Junior	947 (66.8)	0.67 (0.53–0.85)	0.001
Senior	465 (70.7)	0.81 (0.61–1.05)	0.114
Tertiary	429 (73.3)	0.92 (0.70–1.21)	0.553

Factors that were associated with being screened for/counselled on CVDRFs in the univariate model (*p* < 0.05) were then included in a multivariable model adjusting for age, sex and hypertension status. HIV-positive status remained significantly associated with being screened or counselled for CVDRFs in the final multivariable model (AOR 1.84, 95% CI: 1.46–2.32, *p* < 0.001) (Table 4).

**Table 4:** Factors associated with screening and counselling for at least one CVDRF

	Adjusted odds ratio	95% CI	p value
HIV Status*			
Negative	reference		
Positive	1.84	(1.46, 2.32)	< 0.001
**Hypertension status**			
None	reference		
Hypertensive	1.21	(1.15–1.27)	< 0.001
Sex*			
Female	reference		
Male	0.92	(0.89, 0.94)	< 0.001
Age*			
16–24	reference		
25–34	1.1	(1.05, 1.14)	< 0.001
35–44	1.14	(1.09, 1.19)	< 0.001
45–54	1.15	(1.09, 1.22)	< 0.001
55–68	1.17	(1.10, 1.25)	< 0.001
**Level of Education**			
Non-formal	reference		
Primary	1.01	(0.95, 1.06)	0.746
Junior	1.02	(0.96, 1.08)	0.5996
Senior	1.1	(1.03, 1.17)	0.005
Tertiary	1.12	(1.05, 1.19)	0.009

### Subgroup analysis of study population aged 35 years and above

To assess if age is the main factor driving the discrepancies in screening rates for CVDRFs by HIV status, the screening/counselling rates was assessed for participants aged 35 years or older stratified by HIV status (Tables 5, 6 and 7). When excluding persons less than 35 years of age, screening for some of the CVDRFs was not significantly different by HIV status. In particular, diabetes screening, cholesterol screening, salt intake and weight control counsel were not significantly different between PLWH and people without HIV for this sub-population. However, PLWH aged 35 years and above were more likely to have been screened for at least one CVDRF and had significantly higher number of CVDRF screened for and counselled on (Table 5). The adjusted odds ratio (OR) for HIV status were reduced slightly from 1.84 to 1.75.

**Table 5:** Screening and counselling coverage for CVD risk factors by HIV status for participants who are 35 years or older

Counselling/ Screening	ALL n (%)	HIV Infected n (%)	HIV-Uninfected n (%)	p-value
Diabetes screening	596 (28.3)	243 (26.4)	350 (29.6)	0.114
Cholesterol screening	362 (17.2)	161 (17.5%)	191 (16.9%)	0.739
Weight Checked in 3 years	1,402 (66.5)	698 (75.9)	699 (59.2)	< 0.001
Salt intake Counsel	937 (44.4)	405 (44.0)	528 (44.7)	0.787
Smoke Cessation Counsel	173 (59.2)	93 (69.9%)	80 (50.3%)	0.001
Weight control Counsel	781 (37.0)	354 (38.5)	424 (35.9)	0.243
Physical Activity Counsel	904 (43.0)	431 (46.8)	473 (40.1)	0.002
Alcohol Counsel	603 (28.7)	327 (35.5)	276 (23.4%)	< 0.001
One or more CVDRF screened	1,621 (77.2)	748 (81.3)	873 (73.9)	< 0.001
Total of CVDRF screened*	2 (1–5)	3 (1–5)	2 (0–4)	< 0.001

**Table 6:** Univariate sub-analysis of factors associated with screening and counselling for at least one CVDRF for participants aged 35 or above

	Screened/ Counselled for 1 or more risk factors		
	n (%)	Unadjusted Odds ratio (95% CI)	P value
HIV Status*			
Negative (ref)	873 (73.9)	--	
Positive	748 (81.3)	1.53 (1.24, 1.90)	< 0.001
Hypertension *			
None (ref)	1,280 (73.7)		
Hypertensive	347 (93.5)	5.16 (3.44, 8.12)	< 0.001
Sex*			
Female (ref)	1,130 (80.0)	--	
Male	497 (71.5)	0.63 (0.51, 0.78)	< 0.001
**Age**			
35–44 (ref)	668 (76.1)	--	
45–54	460 (76.9)	1.05(0.82, 1.34)	0.709
55–68	499 (79.0)	1.18 (0.92, 1.51)	0.189
**Level of Education**			
Non-formal (ref)	331 (74.9)	--	
Primary	574 (77.0)	1.13 (0.85, 1.48)	0.398
Junior	439 (76.2)	1.07 (0.80, 143)	0.625
Senior	118 (83.7)	1.72 (1.06, 2.88)	0.032
Tertiary	155 (80.7)	1.40 (0.93, 2.15)	0.111

**Table 7:** Factors associated with screening and counselling for at least one CVDRF for participants aged 35 or over

	Adjusted Odds ratio	95% CI	p-value
HIV Status*			
Negative	-		
Positive	1.75	(1.40, 2.19)	< 0.001
**Hypertension status**			
None	-		
Hypertensive	5.23	(3.44, 8.30)	< 0.001
**Sex**			
Female	-		
Male	0.75	(0.60–0.94)	0.010
**Age**			
35–44	reference		
45–54	1.15	(0.86, 1.53)	0.355
55–68	1.27	(0.91, 1.76)	0.157
**Level of Education**			
Non-formal	-	-	
Primary	1.12	(0.83, 1.50)	0.458
Junior	1.31	(0.91, 1.76)	0.142
Senior	2.27	(1.33, 4.01)	0.004
Tertiary	2.03	(1.27, 3.28)	0.003

## Discussion

In this population-based study conducted in a country with a well-established HIV care programme, we demonstrated that while screening and counselling were generally low for each CVDRF, PLWH were overall, more likely to be screened for CVDRFs, including diabetes and hypercholesterolaemia, and more likely to have had their weight checked in the preceding 3 years than those who did not have HIV. Moreover, PLWH were more likely to receive counselling on salt intake, smoking cessation, weight control, physical activity and alcohol consumption than HIV-uninfected participants. This difference persisted among the population aged 35 years and above except for diabetes and hypercholesterolemia screening.

Nearly two-thirds (64%) of the study population was female, which is consistent with data obtained from the national health survey in which 67% of the population was female [26]. The 30% prevalence of HIV in the study population and the high (93%) ART use in this sub-study is consistent with findings of the main BCPP trial [33, 36] but is higher than the national adult HIV prevalence of 18% [37]. The median age of 35 years in the current study could explain the high HIV prevalence, as the national estimate of HIV prevalence among people 35–39 years of age is 42% [37].

Our findings suggest that PLWH were more likely to have received screening for and counselling on CVDRFs than HIV-uninfected participants. This difference persisted after the multivariate analysis that adjusted for age, sex and hypertension status. There are several plausible explanations for this. First, PLWH receive longitudinal outpatient primary care, while most young people without HIV do not receive this care (and rather interact with the health system in cases of illness or pregnancy). Almost all the participants living with HIV were on ART. In the national HIV care program, this results in a minimum of 2 primary care consultations a year with monthly prescription refills, thus increasing the chances of screening and counselling. In contrast, there is no well-established routine primary preventive care program, including on CVDRFs, for those who do not have HIV in Botswana. Second, the knowledge that CVD events occur at higher rates in PLWH than in the general population [38] and a strong association between ART use and CVD mortality risk [39] might have led to more vigilant and systematic screening for CVDRFs in PLWH. Third, some integration of CVDRF screening and counselling into HIV care programmes may explain the difference in the screening and counselling proportions seen between PLWH and the general population [32, 40]. Though the Botswana multisectoral strategy for prevention of NCDs was later adopted in 2018 [30], at the time of the current study, integrated HIV clinical care was already in operation, having been adopted in 2016 [32].

Data on screening and counselling for CVDRFs are generally scant. A study of 175 South African PLWH on antiretroviral therapy demonstrated low screening for and management of CVDRFs, with tobacco smoking included on the list of risk factors screened for [41]. The same study reported that 99.4% of participants had at least one weight measurement in their medical records, with 21.1% and 28.6% of participants being overweight and obese, respectively. However, only 10.9% of the participants reported having been informed of being overweight or obese by their health care provider [41]. The same gap between weight measurement and self-reported behavioural counselling on weight reduction was demonstrated in our current study. The South African study did not compare self-reported screening/counselling by HIV status, and it was conducted among urban PLWH whereas the current study was rural and peri-urban-based.

It is, however, worth noting that the guidelines on the integration of CVD care are not limited to HIV care programmes. Treatment guidelines have recently recommended the integration of CVD screening, management and counselling in primary health care settings [27, 29, 31]. Despite these recommendations, our study showed lower CVDRF screening in the non-HIV population, demonstrating an implementation gap in the management of cardiovascular diseases in Botswana. This is despite the fact that according to the STEPS survey, most adults in Botswana have at least one of the five major risk factors for CVD [26]. Similar findings have been demonstrated in other sub-Saharan countries in which primary health care facilities were not providing services for common noncommunicable diseases such as hypertension, obesity and diabetes despite policies guiding them to do so [42, 43].

Our study current study was conducted prior to the implementation of dolutegravir-containing regimen like tenofovir, lamivudine and dolutegravir (TLD) as first-line for the treatment of HIV. The Botswana integrated HIV clinical care guidelines of 2016 had just come into effect and the wide use of TLD had not taken effect. Since 2016, there has been an uptake and wide use of TLD in the country and with the metabolic concerns associated with its use [44, 45], screening and counselling on CVD risk factors has become even more significant.

### Study limitations

This study has some limitations. First, although this study included a representative random sample of households across Botswana (a strength of this study), most were either rural or peri-urban. Urban areas and remote settlements were not represented, and hence, the sample might not be representative of the entire country. Second, self-reported instead of verifiable documented screening/counselling could have contributed to recall and desirability bias and error. Third, we could not adjust for certain unmeasured potential confounders, including the type of health facility available to the population and the knowledge and experience of health care providers regarding CVDRFs. However, our study did adjust for major confounders (age and sex). Fourth, we did not assess the impact of CVDRF screening and/or counselling on health outcomes.

Despite these limitations, this large, population-based study is one of the first to demonstrate CVDRF screening habits and counselling on these CVDRFs by health care workers in day-to-day health care delivery by HIV status.

## Conclusions

Although Botswana is on track towards achieving the UNAIDS 95-95-95 targets [36] and with it, the integration of screening and counselling for CVDRFs, and having generally adopted CVD management integration strategies in primary health care, this study identified a gap in the screening of the general population for CVDRFs. This discrepancy calls for the strengthening of primary health care systems to provide regular screening, management and counselling on CVDRFs in the general population. The successful HIV care program could serve as a model for integration.

## Data Availability

The data and transcripts used during the study are available from the corresponding author upon reasonable request. The deidentified survey data are available from the Botswana Combination Prevention Project Executive Committee for researchers who meet the criteria for access to confidential data.

## Abbreviations

CVD: Cardiovascular disease
PLWH: People living with HIV
CVDRFs: Cardiovascular disease risk factors
HIV: Human immunodeficiency virus
NCD: Noncommunicable disease
WHO: World Health Organization
WHO PEN: WHO package of essential noncommunicable disease interventions
BP: Blood pressure
IQR: Interquartile range
DALYs: Disability-adjusted life-years
